# Levothyroxine and insulin requirement in autoimmune polyglandular type 3 syndrome: a real-life study

**DOI:** 10.1007/s40618-020-01421-3

**Published:** 2020-10-24

**Authors:** V. Guarnotta, G. Pillitteri, G. Gambino, S. Radellini, E. Vigneri, G. Pizzolanti, C. Giordano

**Affiliations:** Dipartimento di Promozione della Salute, Materno-Infantile, Medicina Interna e Specialistica di Eccellenza “G. d’Alessandro” (PROMISE), Sezione di Malattie Endocrine, del Ricambio e della Nutrizione, Università di Palermo, Piazza delle Cliniche 2, 90127 Palermo, Italy

**Keywords:** Type 1 diabetes mellitus, Autoimmune hypothyroidism, Visceral adiposity index, Irisin, Cardiovascular risk

## Abstract

**Purpose:**

To evaluate factors influencing the insulin and levothyroxine requirement in patients with autoimmune polyglandular syndrome type 3 (APS-3) vs. patients with type 1 diabetes mellitus (T1DM) and autoimmune hypothyroidism (AH) alone, respectively.

**Methods:**

Fifty patients with APS-3, 60 patients with T1DM and 40 patients with AH were included. Anthropometric, clinical and biochemical parameters were evaluated in all patients. Insulin requirement was calculated in patients with APS-3 and T1DM, while levothyroxine requirement was calculated in APS-3 and AH.

**Results:**

Patients with APS-3 showed higher age (*p* = 0.001), age of onset of diabetes (*p* = 0.006) and TSH (*p* = 0.004) and lower total insulin as U/day (*p* < 0.001) and U/Kg (*p* = 0.001), long-acting insulin as U/day (*p* = 0.030) and U/kg (*p* = 0.038) and irisin (*p* = 0.002) compared to T1DM. Patients with APS-3 had higher waist circumference (*p* = 0.008), duration of thyroid disease (*p* = 0.020), levothyroxine total daily dose (*p* = 0.025) and mcg/kg (*p* = 0.006), triglycerides (*p* = 0.007) and VAI (*p* = 0.010) and lower age of onset of thyroid disease (*p* = 0.007) than AH.

At multivariate analysis, levothyroxine treatment and VAI were associated with insulin and levothyroxine requirement in APS-3, respectively. VAI was independently associated with insulin requirement in T1DM. Circulating irisin levels were independently associated with levothyroxine requirement in AH.

**Conclusion:**

Patients with APS-3 show lower insulin requirement and higher levothyroxine requirement than T1DM and AH alone, respectively. Levothyroxine treatment and VAI affect insulin and levothyroxine requirement, respectively, in APS-3. In T1DM, adipose tissue dysfunction, indirectly expressed by high VAI, is associated with an increased insulin requirement, while circulating irisin levels influence the levothyroxine requirement in AH.

## Introduction

Autoimmune polyglandular syndromes (APS) are characterized by the coexistence of at least two autoimmune-mediated endocrinopathies [1–4]. APS type 3 (APS-3) is the most frequent subtype and encompasses type 1 diabetes mellitus (T1DM) and autoimmune thyroid disease and additional non-glandular autoimmune diseases [5, 6]. APS’s are generally characterized by the poor quality of life and increased morbidity and mortality, compared to the single disease and notably to T1DM [7]. The coexistence of T1DM and other autoimmune endocrinopathies impairs glucose metabolism, interferes with effective insulin therapy and deteriorates diabetes control [8]. Among the various factors involved, metabolic control appears to play an important role in the development of micro and macrovascular disease in T1DM, with a great impact on mortality [9, 10].

The daily insulin dose is the cornerstone of the management of T1DM. A low insulin dose generally represents underinsulinization and could cause symptoms of insulin deficiency [11, 12], while a high insulin dose resembles an insulin resistance status [13, 14]. Intra-individual variations in insulin requirements may potentially explain the deterioration of glycaemia once frequent insulin adjustments are not easily available. If drops in insulin needs are indeed considerable and expose patients to bouts of hyperglycaemia, then the therapy’s safety is undermined. The fear of hypoglycaemia may drive patients and care providers to lower the insulin dosage, eventually causing prolonged hyperglycaemia when the insulin needs increase. In addition, the insulin requirement has been also reported to be associated with high cardiovascular risk [15].

The visceral adiposity index (VAI), a gender-specific index based on both anthropometric [body mass index (BMI) and waist circumference (WC)] and metabolic (triglycerides and HDL-cholesterol) parameters, has been proven to be an indicator of both fat distribution and function, correlated with cardiovascular risk [16, 17].

Skeletal muscle is responsible for the majority of insulin-stimulated whole-body glucose disposal; thus, dysregulation of skeletal muscle metabolism can strongly influence whole-body glucose homeostasis and insulin sensitivity and is considered to be the initiating or primary defect that is evident decades before β-cell failure and overt hyperglycaemia develop. Irisin is found to improve insulin resistance by increasing sensitization of the insulin receptor in skeletal muscle and heart by improving hepatic glucose and lipid metabolism, promoting pancreatic β-cell functions, and transforming white adipose tissue to brown adipose tissue [18].

Levothyroxine requirement variations occur depending on the patient’s age, gender, body weight [19, 20] and recently lean body mass has also been shown to be a predictor of the daily requirements for thyroid hormone [21]. Altered thyroid hormones have been described in patients with diabetes especially those with poor glycaemic control. In diabetic patients, the nocturnal TSH peak is blunted or abolished, and the TSH response to TRH is impaired [22]. Interestingly, insulin resistance and β-cell function are inversely correlated with thyroid-stimulating hormone which may be explained by insulin-antagonistic effects of thyroid hormones along with an increase in TSH [23].

The aim of the current real-life observational study was to compare the factors influencing the insulin and levothyroxine (LT4) requirement in patients with autoimmune polyglandular syndrome type 3 (APS-3) compared to T1DM and autoimmune hypothyroidism (AH) alone, respectively.

## Materials and methods

### Subjects

A total of consecutive 150 patients, referred to the Division of Endocrinology of Palermo University from December 2018 to June 2019, were prospectively included in this study. Specifically, 50 patients with APS-3, 14 males and 36 females (mean age 44.7 ± 13.8 years), 60 patients with T1DM, 34 males and 26 females (mean age 36.9 ± 11.3 years) and 40 patients with AH, 8 males and 32 females (mean age 45.1 ± 13.7 years) were included.

Among patients with APS- 3, 43 had the combination of T1DM and AH, while 7 had the combination of latent autoimmune diabetes adults (LADA), 8 had additional vitiligo, 16 had additional celiac disease, 2 had additional hypoparathyroidism, 3 had additional autoimmune hepatitis and 3 had an additional premature ovarian failure. Diagnosis of APS-3 was based on clinical symptoms and serological testing [1]. The diagnosis of T1DM was made according to the ADA guidelines [24], while the diagnosis of AH was made by the presence of antibodies against thyroid peroxidase (TPO antibodies). Among patients with APS-3, 17 were on glargine treatment, 31 were on degludec treatment and 2 were on detemir treatment. In the group of T1DM, 23 were on glargine insulin treatment, 35 were on degludec insulin and 2 were on detemir treatment. Patients with APS and AH were on oral liquid levothyroxine therapy, at the average dose of 1.6 (range 1.4–2) and 1.3 (range 1.2–1.6) mcg/kg, respectively. Patients with APS and celiac disease were on higher levothyroxine dose (1.8–2 mcg/kg) compared to APS without celiac disease. All patients with APS and AH were recommended to postpone breakfast by 30 min following levothyroxine intake.

Patients with celiac disease were on a stable gluten-free diet.

Inclusion criteria were: patients with APS-3, AH and T1DM aged 18–65 years of both genders.

Exclusion criteria were as follows: hypoglycaemia unawareness, acute illnesses or infections, inability to provide informed consent, pregnancy and lactation, duration of thyroid disease less than 3 years and duration of diabetes less than 5 years, subclinical hypothyroidism and thyroid disease not treated with levothyroxine, tablet levothyroxine treatment, presence of known gastrointestinal malabsorption disorders (such as gastric atrophy, helicobacter pylori infection, lactose maldigestion) [25].

All procedures were in accordance with the ethical standards of the responsible committee on human experimentation (institutional and national) and with the Helsinki Declaration of 1964, as revised in 2013. Approval was obtained from the Ethics Committee of the University of Palermo. At the time of hospitalization, an informed consent for the scientific use of the data was obtained from all patients to be included in the study.

All patients included were instructed not to practice physical activity during the two weeks before the biochemical assays. To assess compliance with this instruction, a 7-day pedometer measurement was provided to each patient. Patients with diabetes were trained for strict adherence to carbohydrates counting during one month before biochemical assays.

### Study design

Anthropometric parameters such as BMI, systolic and diastolic blood pressure and waist circumference (WC), measured at the midpoint between the lower rib and the iliac crest, were evaluated in all patients. In addition, lipids [total cholesterol (TC), HDL cholesterol (HDL-C), LDL cholesterol (LDL-C) and triglycerides (TG)], TSH and irisin were obtained in all patients. The insulin requirement was calculated as total daily doses and daily doses per kg of body weight, HbA1c, fasting glycaemia and GOT and GPT were evaluated in patients with APS-3 and T1DM. As an indirect indicator of adipose function and distribution [17], the VAI was calculated in all patients according to gender, where TG levels were expressed in mmol/l and HDL levels were expressed in mmol/l:

Male VAI = [WC/(39.68 + (1.88 × BMI))] × (TG/1.03) × (1.31/HDL); -Fe

male VAI = [WC/(36.58 + (1.89 × BMI))] × (TG/0.81) × (1.52/HDL) [16]

### Assays

Glycaemia, HbA1c and lipids were measured by standard methods (Modular P800, Roche, Milan). LDL-C levels were measured using the Friedewald formula [TC – (HDL + (TG/5)].

Serum samples were analyzed for irisin concentration using a commercial enzyme immunoassay kit (EK-067–29; Phoenix Pharmaceuticals, Karlsruhe, Germany). The lowest detectable concentration of irisin was 9.4 ng/mL, and the highest was 37.5 ng/mL. The kit used in the current study was validated against Western blotting and mass spectrometry [26]. Samples were assayed following the manufacturer’s instructions without a prior extraction step.

The conversion factors for the International System (SI) were as follows: TC and HDL-C mg/dl vs. mmol/l: 0.0259; TG mg/dl vs. mmol/l: 0.0113; HbA1c % vs. mmol/mol: 10.93–23.5.

### Statistical analysis

The SPSS version 19 (SPSS, Inc.) was used for data analysis. Data were presented as mean ± SD or rates and proportions. The normality of distribution of the quantitative variables was assessed using the Kolmogorov–Smirnov test. The differences between the APS-3 and T1DM groups and the difference between the APS-3 and AH groups were evaluated with *t-*Student for quantitative variables and χ^2^ for trend for categorical variables. Relations between the outcome variables and continuous variables were evaluated using univariate Pearson’s correlation coefficients. Multiple linear regression analysis was performed to identify independent predictors of the dependent variables insulin requirements in APS-3 and T1DM and the levothyroxine requirement in APS-3 and AH. The variables added in the multivariate model were those which had a statistical significance at univariate analysis (Pearson correlation). A *p* value of 0.05 was considered statistically significant.

## Results

Patients with APS-3 showed higher age (*p* = 0.001), age of onset of diabetes (*p* = 0.006), VAI (*p* = 0.008) and TSH (*p* = 0.004) and lower total insulin as U/day (*p* < 0.001) and U/Kg (*p* = 0.001) and irisin (*p* = 0.002) compared to T1DM (Table 1).

**Table 1:** General characteristics of patients with autoimmune polyglandular syndrome 3 (APS-3), type 1 diabetes mellitus (T1DM) and autoimmune hypothyroidism (AH)

	APS-3 *No* = *50*	T1DM *No* = *60*	AH *No* = *40*	p*	p**
	Subjects (%)	Subjects (%)	Subjects (%)		
*Gender*					
Males	14 (28%)	36 (60%)	8 (20%)	0.008	0.382
Females	36 (72%)	24 (40%)	32 (80%)		
*Complications*					
Retinopathy	6 (12%)	14 (23.3%)	//	0.127	//
Nephropathy	2 (4%)	5 (8.3%)	//	0.359	//
Neuropathy	2 (4%)	1 (1.6%)	//	0.440	//
Arterial hypertension	9 (15%)	4 (6.6%)	3 (7.5%)	0.152	0.273
Visceral obesity	19 (38%)	15 (25%)	14 (35%)	0.143	0.320
Obesity (BMI ≥ 30 kg/m^2^)	7 (14%)	3 (5%)	12 (30%)	0.103	0.066
Glargine basal insulin	17 ( 34%)	23 (38.3%)	//	0.642	//
Degludec basal insulin	31 (62%)	35 (58.3%)	//	0.694	//
Detemir basal insulin	2 (4%)	2 (3.3%)	//	0.845	//
*Clinical and anthropometric parameters*	Mean ± SD	Mean ± SD	Mean ± SD		
Age (years)	44.7 ± 13.8	36.9 ± 11.3	45.9 ± 13.3	0.001	0.706
BMI (Kg/m^2^)	24.4 ± 4.44	24.5 ± 3.1	25.5 ± 4.76	0.896	0.340
WC (cm)	88.9 ± 8.13	87.5 ± 11.4	96.5 ± 13.1	0.462	0.008
SBP (mmHg)	119.9 ± 17.3	121.1 ± 14.3	115.2 ± 10.9	0.676	0.534
DBP (mmHg)	71.7 ± 9.7	73.7 ± 8.7	70.9 ± 8.3	0.238	0.341
Age of onset of diabetes (years)	25.6 ± 14.8	18.8 ± 9.8	//	0.006	//
Duration of diabetes (years)	17.6 ± 12.3	18.1 ± 11.1	//	0.870	//
Age of onset of thyroid disease (years)	32.7 ± 13.2	//	39.7 ± 13.3	//	0.007
Duration of thyroid disease (years)	12.7 ± 8.36	//	9.44 ± 5.6	//	0.020
*Insulin requirement and glucose control*					
HbA1c (%)	8.04 ± 1.85	8.2 ± 1.37	//	0.501	//
Total insulin/day (U/day)	34.7 ± 12.6	47.8 ± 17.4	//	< 0.001	//
Total insulin/kg (U/kg)	0.54 ± 0.23	0.69 ± 0.24	//	0.001	//
*Levothyroxine requirement*					
Levothyroxine daily dose (mcg/day)	112.6 ± 55.6	//	84.2 ± 33.1	//	0.025
Levothyroxine daily dose/kg (mcg/kg)	1.66 ± 0.79	//	1.24 ± 0.42	//	0.006
*Metabolic parameters*					
Total cholesterol (mmol/L)	4.55 ± 1.12	4.43 ± 1.03	4.91 ± 0.82	0.444	0.173
HDL cholesterol (mmol/L)	1.52 ± 0.41	1.56 ± 0.41	1.37 ± 0.37	0.762	0.123
LDL cholesterol (mmol/L)	2.59 ± 1.03	2.46 ± 0.81	3.18 ± 0.64	0.514	0.240
Triglycerides (mmol/L)	0.93 ± 0.46	0.88 ± 0.75	1.34 ± 0.51	0.852	0.007
GOT (U/L)	23.2 ± 21.1	18.6 ± 6.1	17.6 ± 4.2	0.126	0.856
GPT (U/L)	28.3 ± 22.4	18.8 ± 8.4	19.1 ± 5.6	0.281	0.762
TSH (mU/ml)	3.43 ± 2.46	2.01 ± 0.93	3.25 ± 5.72	0.004	0.436
VAI	1.05 ± 0.45	1.38 ± 1.23	1.88 ± 0.83	0.008	0.001
Irisin (ng/ml)	12.2 ± 2.2	14.1 ± 3.1	13.5 ± 1.8	< 0.001	0.355

Abbreviations: *BMI*: body mass index, *WC* waist circumference, *SBP* systolic blood pressure, *DBP* diastolic blood pressure, *VAI* visceral adiposity index

*Comparison between APS-3 and T1DM

**Comparison between APS-3 and AH

On the other hand, patients with APS-3 had higher WC (*p* = 0.008), duration of thyroid disease (*p* = 0.020), levothyroxine daily dose (*p* = 0.025) and daily dose/kg (*p* = 0.006), triglycerides (*p* = 0.007) and VAI (*p* = 0.010) and lower age of onset of thyroid disease (*p* = 0.007) than AH (Table 1).

Multivariate analysis was done based on the variables who were found to have a statistical significance at univariate analysis (data not shown). It showed that levothyroxine replacement treatment was independently associated with insulin requirement (total daily insulin U/kg) (*p* = 0.044, β =  – 0.005) (Table 2) and the VAI was independently associated with levothyroxine requirement (*p* = 0.035, β = 0.218) in APS-3 (Table 3). In patients with T1DM, the VAI was independently associated with insulin requirement (total daily insulin U/kg) (*p* < 0.001, β = 0.666) (Table 2), while in AH circulating irisin levels were independently associated with levothyroxine requirement (*p* = 0.048, β =  – 0.046) (Table 3).

**Table 2:** Variables independently associated with insulin requirement in patients with autoimmune polyglandular syndrome type 3 (APS-3) and with type 1 diabetes mellitus (T1DM)

Independent variables	Dependent variable total daily insulin (U/kg)			
	T1DM			
	β	*p*	OR	95% CI
Age (years)	– 0.117	0.418	– 0.003	– 0.009 to 0.004
HbA1c (%)	0.058	0.697	0.010	– 0.043 to 0.063
Irisin (ng/mL)	0.452	0.081	–0.032	– 0.057 to 0.008
VAI	0.666	< 0.001	0.152	0.073 to 0.231
TSH (mU/ml)	– 0.033	0.818	– 0.009	– 0.087 to 0.069

Independent variables	Dependent variable total daily insulin (U/kg)			
	APS-3			
	β	*p*	OR	95% CI
Age (years)	– 0.004	0.175	–0.224	–0.009 to 0.002
HbA1c (%)	0.005	0.965	0.008	0.002 to 0.135
Irisin (ng/mL)	0.008	0.617	0.085	–0.023 to 0.038
VAI	0.136	0.411	0.063	–0.092 to 0.219
Levothyroxine treatment	–0.005	0.044	–0.365	–0.249 to 0.239
TSH (mU/ml)	0.210	0.098	0.280	–0.002 to 0.021

Abbreviations: *VAI* visceral adiposity index

**Table 3:** Variables independently associated with levothyroxine requirement in patients with autoimmune polyglandular syndrome type 3 (APS-3) and autoimmune thyroiditis (AH)

Independent variables	Dependent variable: levothyroxine requirement (mcg/kg)			
	APS-3			
	β	*p*	OR	95% CI
Age (years)	0.180	0.811	0.043	– 1.34 to 1.71
HbA1c (%)	– 11.08	0.202	– 0.234	– 28.84 to 6.32
Irisin (ng/mL)	– 0.002	0.967	– 0.007	– 0.116 to 0.111
VAI	0.218	0.035	0.189	0.356 to 1.151
TSH (mU/ml)	0.015	0.502	0.110	– 0.030 to 0.060

Independent variables	Dependent variable: levothyroxine requirement (mcg/kg)			
	AH			
	β	*p*	OR	95% CI
Age (years)	0.038	0.440	0.226	0.001 to 0.075
Irisin (ng/mL)	– 0.046	0.048	-0.745	– 0.212 to 0.120
VAI	0.373	0.251	0.348	– 0.464 to 1.211
TSH (mU/ml)	– 0.268	0.062	-0.632	– 0.562 to 0.026

Abbreviations: *VAI* visceral adiposity index

## Discussion

The current study shows that patients with APS-3 have lower insulin requirement and circulating irisin levels than patients with T1DM and higher levothyroxine requirement and lower VAI than AH.

In patients with APS-3 an inverse correlation between insulin requirement and levothyroxine replacement therapy was found. The relationship between thyroid function and insulin sensitivity has been described in many studies [27–29]. Thyroid hormones enhance gluconeogenesis and glycogenolysis in an opposing effect to insulin [30], whereas they are known to facilitate cellular glucose uptake by expressing the glucose transporter-4 (GLT-4) isozyme [31]. Increased fasting serum insulin levels and lower insulin sensitivity have been reported in hypothyroid patients [32]. Levothyroxine replacement treatment has been shown to reduce fasting and postprandial glucose levels and glycosylated haemoglobin levels in hypothyroidism [27]. Krysiak et al. [33] showed that six-month treatment with levothyroxine does not have a significant effect on HOMA-IR in patients with hypothyroidism. A positive effect of levothyroxine on insulin resistance in patients with euthyroidism is still under investigation. Administration of high doses of thyroid hormones to healthy humans induced insulin resistance [34]. Moderate doses of levothyroxine to healthy subjects lead to short-term mild experimental hyperthyroidism, due to increased peripheral glucose disposal during euglycaemia, which counteracts the increase in splachnic glucose release, and results in maintenance of overall normal glucose tolerance [35]. Small doses of triiodothyronine to lean and obese insulin-resistant rats increased the expression of GLUT4 in skeletal muscle and had a beneficial effect on hyperinsulinaemia [36]. In addition, long-term administration of small doses of levothyroxine to euthyroid horses has been shown to improve glucose dynamics [37]. The impact of administration of small physiological doses of thyroxine to healthy humans in vivo, which suppress TSH within the euthyroid range, has recently been investigated [38]. Lambadiari et al. showed that the insulin-sensitizing effect of thyroid hormone improved glycaemia, insulin sensitivity indices and peripheral glucose uptake and disposal in subjects with type 2 diabetes and that administration of small subthyrotoxic doses of thyroxin to treatment-naive diabetic euthyroid subjects improved glucose disposal in forearm muscle and overall insulin sensitivity, suggesting its therapeutic importance in subjects with insulin resistance by reducing the burden of hyperglycaemia and possibly the long-term complications of diabetes [38].

In the current study, patients with APS-3 also showed lower circulating irisin levels than T1DM. Irisin is a myokine secreted by skeletal muscle and it is strongly involved in metabolism, notably correlated with insulin resistance, as demonstrated by its action in inducing browning of subcutaneous white adipose tissue and activation of thermogenic genes [39, 40]. Many factors affecting circulating irisin levels have been reported both in T1DM and AH alone. However, irisin levels have never been evaluated in patients with APS.

In patients with T1DM, a significant direct correlation between the VAI and insulin requirement was observed, meaning that insulin requirement increases as the VAI increases. Adipose tissue dysregulation (altered fat distribution and function) plays a crucial role in the pathogenesis of insulin resistance and atherosclerosis. Altered adipocytokine production increases in the lipolytic activity of adipose tissue and low-grade inflammation favours visceral obesity and adipose tissue dysfunction. The VAI has been demonstrated to be a good marker of adipose tissue dysfunction. It can express both the altered endocrine function of adipose tissue and the state of relative leptin resistance and low-grade inflammation, which are all alterations of adipose tissue dysfunction [41].

In the current study, patients with T1DM had greater insulin requirement than patients with APS-3 suggesting higher insulin resistance to exogenous insulin. An increased insulin requirement in patients with T1DM and consequently a loss of insulin sensitivity towards exogenous insulin can lead to a phenotype similar to that of subjects with type 2 diabetes mellitus (T2DM) predisposing them to adipose tissue dysfunction. In the current study, a higher VAI in T1DM than APS-3 suggests a worse visceral adipose dysfunction, exposing subjects to increased cardiovascular risk [17].

On the other hand, patients with APS-3 showed a higher levothyroxine requirement than AH and VAI was independently associated with. An association between TSH and adipocytes has been previously reported [41, 42]. TSH has an extra-thyroidal direct effect on adipocytes and elevated TSH levels can promote a proinflammatory response in these cells, by stimulating the release of cytokines, such as IL-6 [43]. Furthermore, studies with TSH-receptor knockout mice have shown that inactivation of the receptor results in the reduced lipolytic effect of TSH and increased adipocyte size, implying that the TSH receptor is important for the growth and metabolism of adipocytes [44, 45]. High TSH may cause the release of adipokines from visceral adipose tissue promoting a pro-inflammatory status that can worsen insulin resistance [44]. However, recently, an interdependent relationship among adipose tissue dysfunction-insulin resistance and thyroid has been suggested. Adipose tissue dysfunction and consequent insulin resistance could reduce type 2 deiodinase activity in the pituitary, reducing intracellular T3 availability in thyrotrophic cells and leading to a TSH increase [44]. The findings of the current study suggest that in patients with APS-3 due to combined T1DM and thyroid disease, the presence of diabetes and adipose tissue dysfunction may lead to an increase of levothyroxine requirement compared to AH alone.

In patients with AH, circulating irisin levels were independently associated with levothyroxine requirement. The skeletal muscle system is an important target of thyroid hormones and is also involved in the production of irisin. During the natural course of AH, the hypothyroidism phase following that of euthyroidism determines reduced production of thyroid hormones and, therefore, the involvement of peripheral target organs. It could be hypothesized that thyroid metabolism may influence circulating irisin levels, probably through muscle damage, which compromises the ability of muscle fibrocells to secrete this myokine [45]. Another hypothesis could be that the reduction of irisin levels in hypothyroid patients could be due to the loss of the stimulating action of fT3 and fT4 on PGC-1α [46].

The current study has some limitations. The sample was quite small and among patients with thyroid disease only those on levothyroxine liquid formulation therapy were evaluated. Some factors impacting insulin doses, such as history and risk of hypoglycaemia, duration of diabetes were not evaluated. In addition, another limitation of this study is that patients with APS-3 followed specific nutritional regimen due to the coexistence of T1DM, while patients with AH did not follow any nutritional prescription and it could have an influence on lipids and WC levels. However, the strength of the study is the parallel evaluation of patients with APS-3, T1DM and AH, that to our knowledge has not been evaluated before.

In conclusion, patients with APS-3 show lower insulin requirement and higher levothyroxine requirement than T1DM and AH alone, respectively. In patients with APS-3, levothyroxine replacement therapy can reduce the insulin requirement, while adipose tissue dysfunction indirectly expressed by VAI can lead to an increased thyroid hormone requirement independently of TSH levels. In patients with T1DM, VAI is associated with insulin requirement, showing that adipose tissue dysfunction leads to an increased insulin requirement. Irisin can be a predictor of levothyroxine requirement in patients with AH. However, further, larger controlled studies are required to confirm our results.
